# Construction Notes

**Published:** 1893-07-22

**Authors:** 


### CONSTRUCTION NOTES.

Willesden Cottage Hospital.—Towards the completion of this hospital, which is being erected on a convenient and healthy site in the centre of Willesden parish, now populated by nearly 70,000 inhabitants, Mr. F. Passmore Edwards, of the *Echo*, has made the handsome gift of £2,000, representing the cost of the building, leaving £1,000 already collected in the district to be invested as the nucleus of an endowment for the support of the institution. Hitherto in the case of accidents it was necessary to take the sufferers to St. Mary's Hospital, nearly four miles distant, and in the case of one of the most recent accidents which proved fatal, the sufferer (whose accident, by a curious coincidence, occurred within a stone's throw of the site of the new hospital) died on his way to St. Mary's. Provision will be made for 20 beds in the new hospital, of which the Duke of Teck has kindly consented to become the first president. The hospital was opened on Tuesday by Miss Balfour, and the Right Hon. A. J. Balfour made an excellent speech on the occasion, thanking Mr. Passmore Edwards for his magnificent gifts on behalf of the people of the district. A flower, fruit, and vegetable show, combined with sports, is announced to be held in August next in further aid of the new hospital.

Convalescent Home at Cromer.—The new convalescent home at Cromer, built and furnished by Mr. B. E. Fletcher, and endowed by the Right Hon. Lord Leicester, K.G., was opened on St. Mark's Day. The house is of red brick, with stone bays and dressings, and is covered with red Broseley tiles. It is designed in the decorated style, with a central tower of three stories. In all the site consists of about three acres, with a lawn at the back of the house, and terraces in front, with balustrades of moulded brick, access being gained from the road by handsome gates. Inside there is evidence that the comfort of the inmates has been studied in all respects, attention having been paid to the ventilation, lighting, and sanitation. In plan the buildings (which were designed by Messrs. Boardman and Son, of Norwich, and executed by Mr. William Chapman, builder, of Hanworth) form an L. The apartments for the women extending along the front, those of the men being in the wing extending backward from the left-hand angle. The isolation block is at the right extremity of the front. To the right of the commodious entrance is the dining hall, 30 ft. by 15 ft.; adjoining and opening into the corridor are the larder, store, and pantry, and on the other side of the passage way are a large scullery, 16 ft. by 15ft., and a kitchen of the same area, also a large store-room. The committee-room, an airy apartment, precedes the matron's parlour. To the right are the lavatories and offices, well lighted and ventilated, and fitted with sanitary appliances of the latest and most approved construction. An excellent children's play-room is also provided, whilst out of the men's day-room leads a smoking-room. A covered way extends along the whole side of the wing. The upper floors are devoted to sleeping rooms and dormitories, lavatories and bath rooms. The isolation block is placed to the extreme right of the front. It is a one-storeyd building, with a ward 16 ft. by 15 ft., the scullery and the offices being in the rear. In all, accommodation is provided for thirty patients, Lord Leicester having contributed £15,000 for endowment.

